# Effect of food on iron absorption in patients with iron deficiency anemia treated with ferric citrate hydrate

**DOI:** 10.1007/s12185-025-04108-8

**Published:** 2025-11-14

**Authors:** Norio Komatsu, Kyoko Ito, Kojo Arita, Yuko Mitobe, Hironori Mitsui

**Affiliations:** 1 Department of Hematology, Juntendo University School of Medicine, 2-1-1, Hongo, Bunkyo-ku, Tokyo 113-8421 Japan; 2 Medical Affairs Department, Torii Pharmaceutical Co., Ltd., 3-4-1, Nihonbashi-Honcho, Chuo-Ku, Tokyo 103-8439 Japan; 3 Clinical Development Department, Pharmaceutical Division, Japan Tobacco Inc., 3-4-1, Nihonbashi-Honcho, Chuo-ku, Tokyo 103-0023 Japan

**Keywords:** Ferric citrate hydrate, Food effect, Iron absorption, Iron deficiency anemia, Crossover study

## Abstract

Iron absorption after the administration of oral iron preparations following a meal is generally reduced compared with fasting. The aim of this study was to investigate the effect of food on iron absorption following ferric citrate hydrate administration in patients with iron deficiency anemia. A randomized, open-label, two-cohort, two-period, single-dose crossover study was conducted to assess the effect of food on iron absorption when 500 mg ferric citrate hydrate (approximately 120 mg of ferric iron) was administered under fasted and fed (immediately after a meal) conditions. Twelve patients aged 20–45 years with iron deficiency anemia (hemoglobin: male 8.0–13.0 g/dL, female 8.0–12.0 g/dL; serum ferritin < 12 ng/mL; transferrin saturation ≤ 16%), participated. The maximum serum iron concentration change was defined as ΔC_max_, and the area under the serum iron concentration change versus time curve from baseline to 24 h after administration as ΔAUC_0-24_. Serum iron levels increased regardless of fasting or fed conditions, and the ΔC_max_ and ΔAUC_0-24_ values were 39% and 29% higher, respectively, under fed versus fasting conditions. No adverse events were reported. In conclusion, food had no notable effect on iron absorption following ferric citrate hydrate administration in patients with iron deficiency anemia.

## Introduction

Iron is indispensable in the body, with the majority attributing to the structure of hemoglobin (Hb) in red blood cells, which transports oxygen throughout the body [1]. Iron is also essential for cell division and proliferation, as well as various metabolic processes [2, 3].

Anemia is characterized by a reduced number of red blood cells or a reduced concentration of Hb in the red blood cells. The main cause of anemia is iron deficiency, which often develops as a result of reduced iron absorption from the small intestine, insufficient iron intake, pregnancy, or chronic blood loss (e.g., during menstruation). Iron deficiency anemia is also common among premenopausal women [4].

Orally ingested iron is absorbed from the upper small intestine and stored mainly in the liver, without being actively excreted. The first-choice treatment for iron deficiency anemia is oral iron preparations. However, if such preparations cannot be tolerated or patients require rapid iron replacement (e.g., prior to scheduled surgery), an intravenous iron preparation is administered [5, 6].

Among the currently marketed oral iron preparations, ferric citrate hydrate (Riona®, Torii Pharmaceutical Co., Ltd, Tokyo, Japan) was originally developed for the treatment of hyperphosphatemia in patients with chronic kidney disease (CKD). Taking advantage of the fact that ferric iron and phosphate form insoluble ferric phosphate following binding [7], ferric citrate (Auryxia®, Akebia Therapeutics Inc., Cambridge, MA, USA), the same active ingredient as that in ferric citrate hydrate (Riona®), was approved for use in patients with dialysis-dependent CKD in the USA in 2014 [8]. In the same year, ferric citrate hydrate (Riona®) was approved for use in patients with dialysis-dependent and non-dialysis-dependent CKD in Japan [9–11].

In previous clinical studies of ferric citrate (Auryxia®) and ferric citrate hydrate (Riona®) as treatment for hyperphosphatemia in patients with CKD, it was shown that serum ferritin and transferrin saturation (TSAT) levels increased, and subsequently the required doses of erythropoiesis-stimulating agents for the treatment of renal anemia and intravenous iron preparations decreased [12, 13].

Based on these results, further clinical studies were conducted to evaluate the efficacy and safety of iron replacement of these ferric compounds in patients with iron deficiency anemia [14, 15]. Consequently, ferric citrate (Auryxia®) and ferric citrate hydrate (Riona®) were approved for the treatment of iron deficiency anemia as an additional indication in patients with non-dialysis-dependent CKD in the USA in 2017, and for patients with iron deficiency anemia in Japan in 2021.

In general, iron absorption after taking oral iron preparations is lower under fed conditions compared with fasted conditions. Indeed, after treatment with ferrous sulfate or ferrous fumarate, iron absorption under fed conditions was lower than under fasted conditions in patients with iron deficiency [16, 17]. Furthermore, reduced iron absorption was reported when a single dose of ferrous sulfate or ferric maltol (Feraccru®, Shield Therapeutics Ltd, Gateshead UK) was administered to patients with iron deficiency anemia along with a meal compared with under fasting conditions [18]. Therefore, food has a significant impact on iron absorption of oral iron preparations.

Based on the characteristics of other oral iron preparations, we designed this study to evaluate the food effect on iron absorption following ferric citrate hydrate administration in patients with iron deficiency anemia.

## Materials and methods

### Study design and study treatment

This food effect study was a randomized, open-label, two-cohort, two-period, oral single-dose crossover clinical pharmacology study to evaluate the impact of food on iron absorption after administration of ferric citrate hydrate in patients with iron deficiency anemia.

**Fig. 1:**
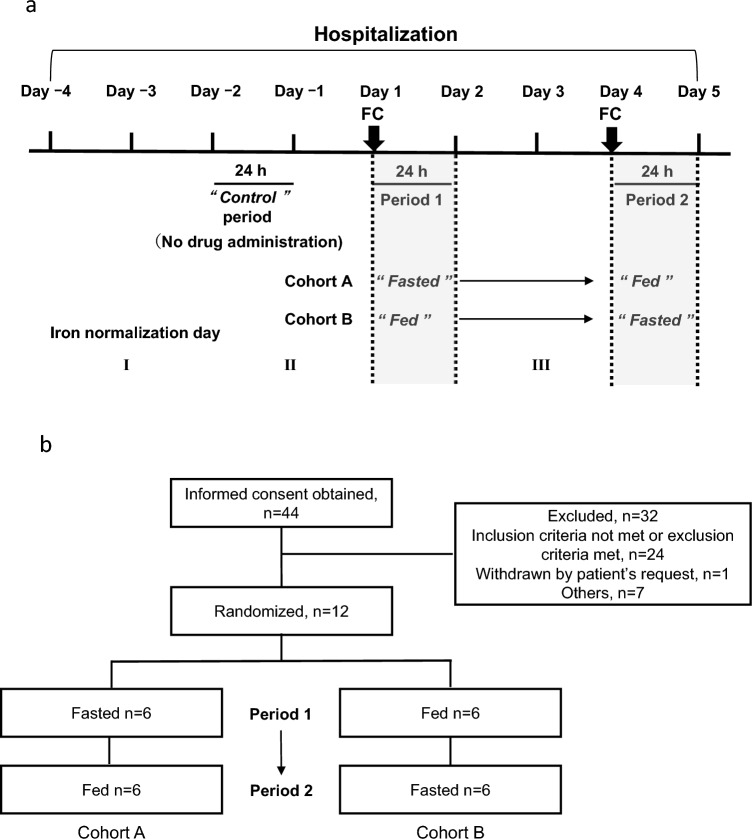
Study schema (**a**) and patient disposition (**b**). *FC* ferric citrate hydrate

The study period (days ˗4 to 5) consisted of a hospitalization day (day ˗4), iron normalization day I (day ˗3), control period (day ˗2 to day ˗1, no study drug administration), iron normalization day II (day ˗1), period 1 study drug administration day and the follow-up day (day 1 to day 2), iron normalization day III (day 3), and period 2 study drug administration day and the follow-up day (day 4 to day 5) with 72 h between the period 1 and period 2 administration days (Fig. 1a). The iron normalization days were set so that patients had the same meals and the effect on serum iron concentration measurement the following day could be standardized. The control period was included in the study design to confirm the fluctuations of serum iron concentrations and effect of food intake without administration of ferric citrate hydrate. Patients were discharged from the hospital after completing all examinations one day after administering ferric citrate hydrate in period 2 (day 5).

Patients were randomized into two cohorts: cohort A, in which patients were treated with ferric citrate hydrate under fasting conditions in period 1 (day 1 to day 2) and fed conditions (immediately after a meal) in period 2 (day 4 to day 5); and, cohort B, in which patients were treated with ferric citrate hydrate under fed conditions (immediately after a meal) in period 1 and under fasting conditions in period 2.

On the days that ferric citrate hydrate was administered (day 1 in period 1 and day 4 in period 2), patients were treated with ferric citrate hydrate at 09:00, either 10 min after breakfast (under fed conditions) or without breakfast (under fasted conditions). When patients were administered ferric citrate hydrate under fasted conditions, they were instructed to refrain from eating until lunchtime. In each period, patients were administered two tablets of 250 mg ferric citrate hydrate (500 mg ferric citrate hydrate with approximately 120 mg of elemental ferric iron) with water. All meals were prepared so that daily iron intake was ≤ 5.0 mg [19], in order to minimize the impact of iron contained in the meals during hospitalization, except on day ˗4, and other nutrients were controlled according to the Dietary Reference Intakes for Japanese (2015) [20], which recommends an ascorbic acid intake of 100 mg per day. Patients had the same meals during the control period (day ˗2) and the study drug administration days in period 1 (day 1) and period 2 (day 4), so that the food effect on measurements of serum iron concentrations (24 h) were normalized. Patients were advised to drink only water from the time they woke up until 13:00 on day ˗2 (control period)) and until 4 h after the study drug administration on day 1 (period 1) and day 4 (period 2).

The concomitant use of any medications or therapies was prohibited during hospitalization.

### Patients

This study included Japanese patients with iron deficiency anemia, aged ≥ 20 and ≤ 45 years old, with a Hb level ≥ 8.0 and < 13.0 g/dL in males and ≥ 8.0 and < 12.0 g/dL in females, serum ferritin < 12 ng/mL, TSAT ≤ 16%, and a body mass index ≥ 16.0 and < 25 kg/m^2^.

Key exclusion criteria were as follows: patients with anemia caused by a factor other than iron deficiency; premenopausal women who were expecting menstruation to start during the scheduled hospitalization, or with abnormal bleeding within 4 weeks prior to hospitalization; positive for *Helicobacter pylori* antibody; patients with infections or disorders with obvious gastrointestinal bleeding; patients with gastrointestinal diseases such as acute peptic ulcer, chronic colitis ulcerative, or regional enteritis (excluding chronic gastritis); paroxysmal nocturnal hemoglobinuria; hypersensitivity to preparations containing iron; a history of gastric or duodenectomy (excluding endoscopic resection such as polypectomy); treatment with intravenous or oral iron formulae for iron within 4 weeks prior to hospitalization; treatment with erythropoietin stimulating agents, anabolic hormones, testosterone enanthate, mepitiostane, or blood transfusion within 16 weeks prior to hospitalization; blood collection of > 400 mL within 12 weeks before hospitalization, > 200 mL within 4 weeks before hospitalization, or apheresis donation within 2 weeks before hospitalization; or past treatment with ferric citrate hydrate.

### Outcomes and assessments

In this study, serum iron concentrations were evaluated as a pharmacokinetic outcome, and treatment-emergent adverse events (TEAEs), adverse drug reactions (ADRs), vital signs, a standard 12-lead electrocardiogram (ECG), and clinical examinations were evaluated as safety outcomes.

In addition, serum ferritin, total iron-binding capacity (TIBC), and TSAT levels were evaluated. Other assessments included the status of food intake (either complete or incomplete) and menstruation (female patients only).

Blood samples were collected on days ˗4, ˗2, ˗1, 1, 2, 4, 5, and at the time of study discontinuation. For the pharmacokinetic analyses, blood was drawn ˗1, 1, 2, 3, 4, 6, 8, and 12 h after administration of ferric citrate hydrate on day 1 and day 4. For the control, blood samples were obtained at the same time points without administration of ferric citrate hydrate on day ˗2.

### Analytical methods

The sample size was set at six patients in each cohort, with a total of 12 patients included to evaluate the effects of food on iron absorption and safety.

Patients, who had at least one pharmacokinetic examination within 24 h of ferric citrate hydrate administration in each period, were included in the pharmacokinetic analysis set. Patients, who were treated with ferric citrate hydrate and had at least one safety examination, were included in the safety analysis set.

The baseline values for pharmacokinetics and other investigation items were defined as follows: 1 h before administration of ferric citrate hydrate in fed and fasted conditions, and 1 h before the base point in the control. For the analysis of safety, baseline values were obtained on day 1 and before administration of ferric citrate hydrate.

Descriptive statistics were calculated for baseline patient characteristics by cohort and in all patients in the safety analysis set.

The following pharmacokinetic parameters were calculated based on the serum iron concentrations using the non-compartment model: time to maximum concentration (t_max_), maximum concentration (C_max_), terminal half-life (t_1/2_), maximum concentration change (ΔC_max_), area under the concentration–time curve from baseline to 24 h (AUC_0–24_), and area under the concentration change versus time curve from baseline to 24 h after administration (ΔAUC_0–24_). For pharmacokinetic parameters and serum iron levels, descriptive statistics (sample size, mean, standard deviation [SD], median with range, coefficient of variation [%], and geometric mean and 95% confidence interval [CI]) were calculated in fed and fasted conditions, except the geometric mean and the 95% CI for the t_max_ and t_1/2_.

The effects of food were examined using ΔC_max_ and ΔAUC_0–24_ obtained under fasting and fed conditions, as changes from baseline values. The least-squares means and 90% CIs were calculated for the logarithmically transformed ΔC_max_ and ΔAUC_0–24_ using the linear mixed effects model.

TEAEs were recorded after administration of ferric citrate hydrate in period 1 until patients were discharged, and were coded according to the Medical Dictionary for Regulatory Activities (MedDRA/J V19.1).

Imputation of missing data was not performed. Statistical analyses were carried out using SAS version 9.3 or later (SAS Institute Inc., Cary, NC, USA).

This study was conducted in compliance with the Declaration of Helsinki and Good Clinical Practice guidelines. The study and related documents such as the study protocol and informed consent form were approved by the institutional review boards. Prior to study participation, written informed consent was obtained from all patients. The study has been registered at the Japan Registry of Clinical Trials (jRCT2080223447).

## Results

### Patients and baseline characteristics

This study was conducted from January 26 to May 2, 2017 at one study site.

A total of 44 patients provided written consent, of whom, 12 patients were randomized into two cohorts (Fig. 1b). Six patients each were assigned to the fasted first cohort A and fed first cohort B in period 1, who were then switched to the fed and fasted cohorts in period 2, respectively. All 12 patients completed the entire study.

All 12 patients took breakfast completely. None of the female patients in the study reported menstruation from the control period to 24 h after period 2 administration day.

The mean ± SD age of the 12 patients was 32.4 ± 8.0. Of the 12 patients, 9 (75.0%) were female, all of whom were premenopausal. The primary disease resulting in iron deficiency anemia was unknown in 9 (75.0%) patients and menorrhagia in 3 (25.0%) patients (Table 1).

**Table 1:** Demographics and baseline characteristics (safety analysis set)

	Cohort A Fasted first (n = 6)	Cohort B Fed first (n = 6)	Total (n = 12)
Age (years)	32.7 ± 8.4	32.2 ± 8.4	32.4 ± 8.0
Sex			
Male	2 (33.3%)	1 (16.7%)	3 (25.0%)
Female	4 (66.7%)	5 (83.3%)	9 (75.0%)
Menopausal status (women only)			
Premenopausal	4 (100.0%)	5 (100.0%)	9 (100.0%)
Height (cm)	160.30 ± 6.18	160.25 ± 10.78	160.28 ± 8.38
Body weight (kg)	54.72 ± 8.25	52.18 ± 5.55	53.45 ± 6.83
BMI (kg/m^2^)	21.31 ± 3.07	20.36 ± 1.69	20.84 ± 2.41
Systolic blood pressure (mmHg)	113.2 ± 8.5	105.0 ± 12.4	109.1 ± 11.0
Diastolic blood pressure (mmHg)	62.3 ± 4.6	62.5 ± 8.9	62.4 ± 6.7
Pulse rate (beats/min)	66.3 ± 10.6	59.2 ± 11.9	62.8 ± 11.3
Body temperature (°C)	36.55 ± 0.20	36.47 ± 0.23	36.51 ± 0.21
Medical history			
None	6 (100.0%)	6 (100.0%)	12 (100.0%)
Comorbidities			
None	6 (100.0%)	6 (100.0%)	12 (100.0%)
Prior treatment with iron supplements			
Yes			
Oral	1 (33.3%)	2 (66.7%)^a^	3 (50.0%)^a^
IV	2 (66.7%)	2 (66.7%)^a^	4 (66.7%)^a^
No	3 (50.0%)	3 (50.0%)	6 (50.0%)
Primary disease			
Menorrhagia	1 (16.7%)	2 (33.3%)	3 (25.0%)
Unknown	5 (83.3%)	4 (66.7%)	9 (75.0%)

Data are presented as the mean ± SD or *n* (%)

*BMI* body mass index; *IV* intravenous; *SD* standard deviation

^a^Patients treated with both oral and IV iron supplements are included

Baseline values of iron-related parameters are summarized in Table 2. The mean ± SD baseline levels of serum iron (24.7 ± 5.9 μg/dL under fasted conditions, 25.5 ± 7.4 μg/dL under fed conditions, and 28.2 ± 8.7 μg/dL in the control), serum ferritin (5.80 ± 1.27 ng/mL, 6.27 ± 2.25 ng/mL, and 5.99 ± 1.16 ng/mL), TIBC (410.5 ± 32.9 μg/dL, 409.6 ± 33.8 μg/dL, and 410.2 ± 33.5 μg/dL), and TSAT (6.00% ± 1.25%, 6.23% ± 1.70%, and 6.84% ± 1.91%) showed no notable differences in the fasted, fed, and control groups.

**Table 2:** Baseline values of pharmacokinetic and iron-related parameters (pharmacokinetic analysis set)

	Fasted (n = 12)	Fed (n = 12)	Control (n = 12)
Serum iron (µg/dL)	24.7 ± 5.9	25.5 ± 7.4	28.2 ± 8.7
Serum ferritin (ng/mL)	5.80 ± 1.27	6.27 ± 2.25	5.99 ± 1.16
TIBC (µg/dL)	410.5 ± 32.9	409.6 ± 33.8	410.2 ± 33.5
TSAT (%)	6.00 ± 1.25	6.23 ± 1.70	6.84 ± 1.91

Data are presented as the mean ± SD

*SD* standard deviation; *TIBC* total iron-binding capacity; *TSAT* transferrin saturation

### Serum iron and its pharmacokinetic parameters

**Fig. 2:**
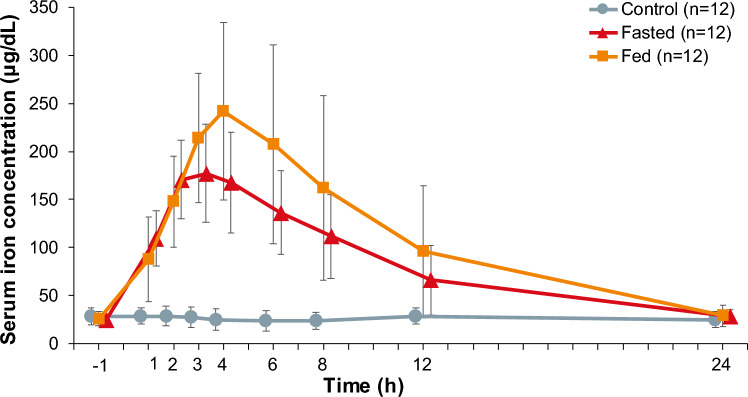
Time course changes in serum iron concentrations. The results are presented as the mean ± standard deviation (n = 12)

The serum iron concentration increased over time after a single dose of 500 mg ferric citrate hydrate under both fasted and fed conditions (Fig. 2), whereas the serum iron concentration did not change over 24 h post-administration in the control.

When a single oral dose of 500 mg ferric citrate hydrate was administered under fasted conditions, the serum iron concentration also increased, reaching a C_max_ of 182.4 ± 46.7 μg/dL and a t_max_ of 2.500 h (median, range 1.00–3.00) after administration (Table 3), and then decreased with a t_1/2_ of 5.340 ± 2.290 h. Under fed conditions, the mean ± SD serum iron concentration increased, reaching a C_max_ of 250.8 ± 86.9 μg/dL and a t_max_ of 4.000 h (median, range 2.00–6.00) after administration, and then decreased with a t_1/2_ of 5.270 ± 0.759 h (Table 3). No notable changes were observed in the control (Table 3).

**Table 3:** Descriptive statistics of pharmacokinetic parameters (pharmacokinetic analysis set)

	Fasted (n = 12)	Fed (n = 12)	Control (n = 12)
t_max_ (h)^a^	2.500 (1.00–3.00)	4.000 (2.00–6.00)	3.000 (0.00–24.00)
t_1/2_ (h)	5.340 ± 2.290^b^	5.270 ± 0.759^c^	–^d^
C_max_ (μg/dL)	182.4 ± 46.7	250.8 ± 86.9	31.6 ± 10.1
ΔC_max_ (μg/dL)	157.8 ± 48.0	225.3 ± 86.2	3.4 ± 4.0
AUC_0–24_ (μg·h/dL)	1971.6 ± 658.3	2548.9 ± 1190.5	624.9 ± 198.7
ΔAUC_0–24_ (μg·h/dL)	1342.8 ± 654.6	1883.8 ± 1126.1	˗50.5 ± 63.5

Data are the mean ± SD unless otherwise specified

*AUC*_*0–24*_ area under the concentration–time curve from baseline to 24 h; *ΔAUC*_*0–24*_ area under the concentration change versus time curve from baseline to 24 h after administration; *C*_*max*_ maximum concentration; *ΔC*_*max*_ maximum concentration change; *SD* standard deviation; *t*_*1/2*_ terminal half-life; *t*_*max*_ time to maximum concentration

^a^Median (min–max)

^b^*n* = 3

^c^*n* = 6

^d^Not calculated

The geometric least-squares mean ratios of serum iron after ferric citrate hydrate administration under fed conditions compared with fasting conditions were 1.39 (90% CI: 1.17–1.64) μg/dL for ΔC_max_ and 1.29 (90% CI: 0.96–1.74) μg·h/dL for ΔAUC_0–24_ (Table 4).

**Table 4:** Analysis results of the food effect on iron absorption following administration of ferric citrate hydrate (pharmacokinetic analysis set)

	Geometric LSM		Ratio of geometric LSMs in the fed vs fasted (90% CI)
	Fasted (n = 12)	Fed (n = 12)	
ΔC_max_ (μg/dL)	151.3	209.6	1.39 (1.17–1.64)
ΔAUC_0–24_ (μg·h/dL)	1199.8	1548.2	1.29 (0.96–1.74)

ΔAUC_0–24_, area under the concentration change versus time curve from baseline to 24 h after administration

*CI* confidence interval; *ΔC*_*max*_ maximum concentration change; *LSM* least-squares mean

### Other iron-related parameters

**Fig. 3:**
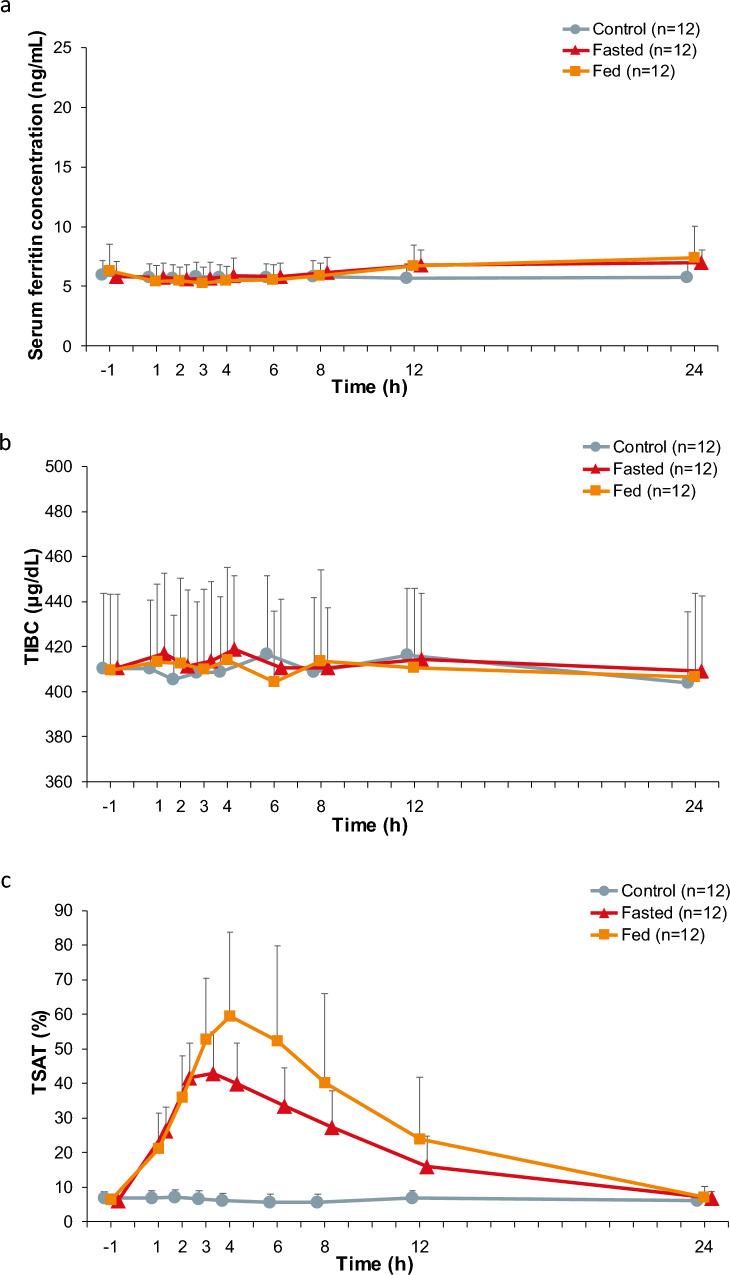
Time course changes in (**a**) serum ferritin (ng/mL), (**b**) TIBC (μg/dL), and (**c**) TSAT (%). The results are presented as the mean + standard deviation (n = 12.). *TIBC* total iron-binding capacity; *TSAT* transferrin saturation

No notable changes in serum ferritin and TIBC levels were observed after administration of ferric citrate hydrate irrespective of feeding conditions (i.e., fasting or fed) (Fig. 3a, b). By contrast, the mean ± SD of the TSAT level increased from baseline (6.00 ± 1.25%), reaching 42.95 ± 11.95% at 3 h after administration of a single dose of 500 mg ferric citrate hydrate under fasted conditions (Fig. 3c). Similarly, under fed conditions, the TSAT level at baseline was 6.23 ± 1.70%, which increased after administration of ferric citrate hydrate, reaching 59.42 ± 24.31% at 4 h after administration, and declining thereafter. No notable changes were observed in the control.

### Safety

No TEAEs or ADRs were observed in this study, and no clinically meaningful changes in the clinical examinations, vital signs, or ECG results were observed irrespective of feeding conditions.

## Discussion

In this study, the food effect on iron absorption of a single oral dose of ferric citrate hydrate 500 mg was examined in patients with iron deficiency anemia. The results demonstrated an increase in serum iron concentration after administering ferric citrate hydrate under both fasting and fed conditions. After the administration of ferric citrate hydrate, the ΔC_max_ and ΔAUC_0–24_ values under fed conditions were higher than under fasted conditions by 39% and 29%, respectively. It is speculated that the prolonged gastric retention time of iron caused by the meal [21, 22], together with increased splanchnic blood flow induced by the meal [23] resulted in a longer intestinal transit time and increased iron availability through at the absorption sites in the upper small intestine. This finding indicated that food did not reduce the iron absorption of ferric citrate hydrate. Furthermore, the time taken to reach maximum serum iron concentration from baseline and the peak concentrations in the fasted and fed conditions indicated that food had no significant impact on the absorption of iron after the administration of ferric citrate hydrate. These results suggested that there were no clinically meaningful changes on iron absorption of ferric citrate hydrate between fasted and fed conditions.

Iron absorption was evaluated based on changes in serum iron levels, because a positive correlation has been reported between the increase in serum iron concentration and Fe-labeled iron absorption when used oral iron preparation in patients with iron deficiency anemia [24].

For oral iron preparations, solubilization of the iron at the low pH of gastric acid is key to the iron absorption process [25, 26]. The solubilized ferrous iron is absorbed in the upper small intestine, while ferric iron is reduced to ferrous iron before being absorbed. It has been demonstrated that the combined administration of oral ferrous sulfate with a gastric acid secretion inhibitor (omeprazole), which increases the pH in the stomach [27], did not result in an increased serum iron concentration [28]; however, the serum iron concentration did increase after the discontinuation of omeprazole treatment [28]. Similarly, food increases the pH in the stomach [29]. Therefore, it is assumed that iron solubility is reduced and iron absorption is attenuated under fed conditions (high pH in stomach) compared with fasting conditions (low pH in stomach).

**Fig. 4:**
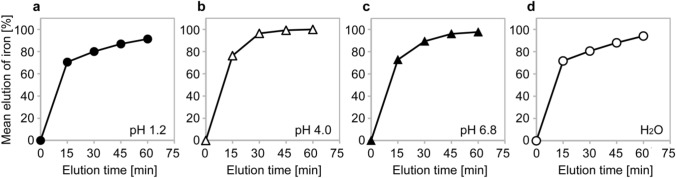
Elution behavior of iron from ferric citrate hydrate (Riona®) under different pH conditions. The elution solution was filtered through a 0.45-μm membrane filter, followed by reduction using L-ascorbic acid. The iron concentration was determined by the 1,10-phenanthroline spectrometric method and the elution rate was calculated using a standard solution of iron (1000 mg/L Japan Calibration Service System), which was used as a 100% elution solution. The mean elution ratio of solutions adjusted to (**a**) pH 1.2, (**b**) pH 4.0, (**c**) pH 6.8, and (**d**) a water control (*n* = 6 for each). Cited from Ito K, et al. Int Urol Nephrol. 2023; 55:141–50 [31] (https://creativecommons.org/licenses/by/4.0/), modified from Koyama J, et al. (Jpn J Clin Dialysis 2015; 31:1543–9 [27]), with permission granted by the journal

As mentioned earlier, absorption of oral iron preparations is greatly affected by the pH in the stomach. However, as shown in an in vitro study, the active ingredient of ferric citrate hydrate dissolves over a wide range of pH values (Fig. 4) [30]. Therefore, it is speculated that ferric citrate hydrate dissolves irrespective of pH in the stomach, even when it is administered immediately after a meal (high pH in stomach). Ferric iron becomes insoluble at pH > 3 unless it is chelated after dissolution [31]. After dissolution, ferric citrate hydrate may have formed chelate complexes, namely ferric citrate complexes, from citric acid and ferric iron [32, 33], which remain soluble regardless of pH. Then, ferric iron in the chelate complexes is assumed to be reduced to ferrous iron and absorbed from the upper small intestine.

A study of CKD patients with hyperphosphatemia who were treated with ferric citrate hydrate immediately after meals for 12 weeks and 52 weeks, revealed that the serum ferritin and TSAT levels increased regardless of the concomitant use of gastric acid secretion inhibitors [34], endorsing the possibility that ferric citrate hydrate is dissolved, chelated, and absorbed across a wide range of pH values in the stomach. These findings are consistent with the results of the present study.

The TSAT level increased, consistent with the trend in serum iron concentration after administration of ferric citrate hydrate. The TSAT is one of the markers to evaluate iron deficiency and the level indicates the iron availability for erythropoiesis [5], and these results therefore suggest that the absorbed iron rapidly bound to transferrin following the administration of ferric citrate hydrate and was utilized in erythropoiesis in patients with iron deficiency anemia.

This study has several limitations. Firstly, the sample size may not have been sufficient to assess the frequency of adverse events between the fasting and fed conditions. Secondly, the chelate complex of ferric citrate hydrate itself has not been identified. Accordingly, the actual mechanism by which food did not reduce the iron absorption of ferric citrate hydrate remains unclear. Finally, iron absorption is known to be inhibited by tannins, phytic acid, polyphenols, calcium, oxalic acid, and enhanced by ascorbic acid, citric acid present in meals [22, 35, 36]. The impact of these meal components on iron absorption was not investigated in this study.

In conclusion, the results of this study demonstrate an increase in serum iron levels under both fasting and fed conditions, and no decrease in iron absorption due to food. There is therefore no apparent clinically meaningful food effect on iron absorption following ferric citrate hydrate administration in patients with iron deficiency anemia.

## Data Availability

All data supporting the findings of this study are available within the paper.
